# Recent Advances in Computational Modeling of Biomechanics and Biorheology of Red Blood Cells in Diabetes

**DOI:** 10.3390/biomimetics7010015

**Published:** 2022-01-13

**Authors:** Yi-Xiang Deng, Hung-Yu Chang, He Li

**Affiliations:** 1 School of Engineering, Brown University, Providence, RI 02912, USA; yixiang_deng@brown.edu; 2 Division of Applied Mathematics, Brown University, Providence, RI 02912, USA; hyc.brown@gmail.com; 3 Center for Biomedical Engineering, Brown University, Providence, RI 02912, USA

**Keywords:** computational modeling, diabetes mellitus, red blood cell, RBC deformation, RBC aggregation, blood rheology

## Abstract

Diabetes mellitus, a metabolic disease characterized by chronically elevated blood glucose levels, affects about 29 million Americans and more than 422 million adults all over the world. Particularly, type 2 diabetes mellitus (T2DM) accounts for 90–95% of the cases of vascular disease and its prevalence is increasing due to the rising obesity rates in modern societies. Although multiple factors associated with diabetes, such as reduced red blood cell (RBC) deformability, enhanced RBC aggregation and adhesion to the endothelium, as well as elevated blood viscosity are thought to contribute to the hemodynamic impairment and vascular occlusion, clinical or experimental studies cannot directly quantify the contributions of these factors to the abnormal hematology in T2DM. Recently, computational modeling has been employed to dissect the impacts of the aberrant biomechanics of diabetic RBCs and their adverse effects on microcirculation. In this review, we summarize the recent advances in the developments and applications of computational models in investigating the abnormal properties of diabetic blood from the cellular level to the vascular level. We expect that this review will motivate and steer the development of new models in this area and shift the attention of the community from conventional laboratory studies to combined experimental and computational investigations, aiming to provide new inspirations for the development of advanced tools to improve our understanding of the pathogenesis and pathology of T2DM.

## 1. Introduction

Diabetes mellitus, coined for chronically elevated blood glucose levels, is a metabolic disease affecting about 29 million Americans and more than 422 million adults all over the world [1]. It is recognized as the world’s fastest-expanding epidemic, among which type 2 diabetes mellitus (T2DM) takes up over 90% of the cases of vascular diseases and its prevalence is increasing due to the rising obesity rates in modern societies [2]. People with T2DM experience an increased risk of developing cardiovascular (CVD) and cerebrovascular diseases (CeVD) [3]. Formation of atherosclerotic plaques, a critical step of atherosclerosis, is a very slow process (years to decades) characterized by sub-endothelial accumulation of leucocytes, mostly macrophages, lipids, calcium, debris, and fibrous connective tissue [4]. The accumulated material increases wall thickness, which may intrude into the artery lumen, hence restricting blood flow. When the atherosclerotic plaque ruptures, the plaque cap recruits platelets through von Willebrand factor and forms a stable or unstable clot with the help of circulating coagulation factors, fibrin, and red blood cells (RBCs). Blood clot formation after the plaque ruptures is an acute event, which can severely reduce or completely block blood flow in large vessels in a few minutes (thrombosis). In addition, blood clots could form in a vein located deep inside the human body. The venous thrombus is less stable than the thrombus formed in the arteries such that part of the clot often detaches (emboli) and flows in circulation, potentially causing blockage of the blood flow in arteries of smaller diameter (thrombo-embolism) [4].

In addition to macrovascular diseases, T2DM is associated with many microvascular diseases, like retinopathy, nephropathy, and neuropathy [5], which result from the persistent hyperglycemia and the abnormal rheological properties of RBCs and platelets. The compromised blood microcirculation, because of the lasting rouleaux structures and modified RBC membrane properties, imposes a huge risk on the functionality of cells and tissues. Hence, studying blood cell dysfunction and quantifying RBC aggregation and disaggregation dynamics under normal or diseased metabolism is of fundamental importance in diabetic research [6]. Previous studies have demonstrated that both plasma fibrinogen in vivo and dextran in vitro are positively correlated to the rouleaux formation [7,8,9,10,11]. While the fibrinogen molecule is considered to be the major plasma protein promoting RBC rouleaux formation [7], high levels of synthetic dextran can also increase the formation of RBC aggregates [8,9,10,11]. Along this line, several in-vitro studies have been performed to quantify the adhesive forces between diabetic RBCs [8,12,13,14,15,16,17,18,19,20,21,21] using various experimental techniques, such as microfluidics, optical tweezers (OT), and atomic force microscopy (AFM).

In the last two decades, numerous computational models have been developed, using either particle-based approaches or continuum-based algorithms [22,23,24,25,26,27,28,29,30], to simulate the dynamics of RBCs under static and flow conditions. While in the particle-based methods, such as dissipative particle dynamics (DPD), blood cell models are constructed using DPD particles and thus are naturally assimilated with the background flow, continuum-based RBC models often implemented a boundary integral algorithm or the immersed boundary method (IBM) to couple RBC models with the background flow, which are solved using different solvers, including the finite volume method, the finite element method and the lattice Boltzmann method [31,32,33,34,35,36,37,38,39,40,41]. Motivated by experimental studies, recent progress in computational modeling has enabled simulations of fluid dynamics and cell aggregation dynamics under physiological and pathological states [10,27,42,43,44,45,46,47,48,49,50], which has provided insight into the pathogenesis of the disease as well as facilitated the development of therapeutic treatments [51,52]. For example, RBC models developed using DPD are widely applied to simulate the deformation and aggregation of healthy RBC doublets [42] together with their effects on the blood cell dynamics in stenosed microvessels [43]. Investigations of the rouleaux formation under diseased conditions have also been conducted [27,45,46,53] and these works provide important observations that cannot be detected directly from experimental studies. In this review, we are going to focus on the computational studies of biomechanics and biorheology of RBCs in T2DM. Specifically, we will review the recent advances in modeling diabetic blood from the single-cell level to the blood flow in microvessels, including the biomechanics of single RBCs (Section 2), enhanced adhesion of diabetic RBCs (Section 3), biorheology of diabetic blood, and platelet margination (Section 4) as well as platelet aggregation in diabetic blood (Section 5), respectively.

## 2. Aberrant Biomechanics and Morphologies of Diabetic RBCs

**Figure 1:**
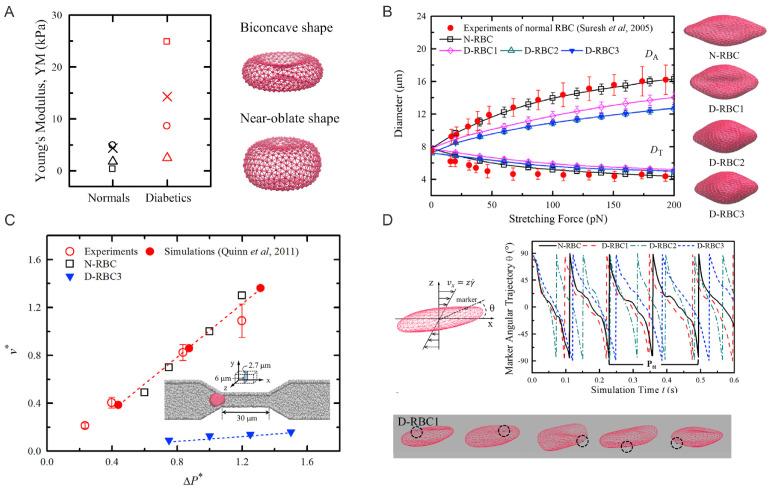
(**A**) Left: Young’s moduli of normal and diabetic RBCs reported from Fornal et al. [61] (crosses); Ciasca et al. [62] (triangles); Zhang et al. [63] (squares); Lekka et al. [64] (circles). Right: Particle-based models for RBCs with equilibrium biconcave (A/V = 1.44) and near-oblate (A/V = 1.04) shapes. (**B**) Comparison of stretching responses between normal and three T2DM RBC models. Experimental data by Suresh et al. [65], and stretching force at 100 pN are shown next to the figure. (**C**) Normal and diabetic RBC traversing velocities ($v^{*}$) in a microfluidic channel with a width of 6.0 μm under difference pressure gradients (▵P). Experimental and simulation results from prior work of Quinn et al. [66] are plotted for comparison. (**D**) Tank-treading motion of normal and diabetic RBCs in Couette flow. Top: angular trajectories of marked particles on the cell membrane during the TT motion for different RBC models at a shear rate 105 s${}^{-1}$ are plotted. Bottom: Snapshots of diabetic RBC model at time *t* = 0.20 s, 0.25 s, 0.30 s, 0.35 s, and 0.40 s. Figures are adopted from [58] with permission.

For RBCs in pathological states, the changes in cell morphology and mechanical properties of RBCs are believed to be responsible for the undermined functionality including loss of deformability. Decreased RBC deformability has been confirmed in T2DM. Experimental studies using micropipette aspiration and filtration methods [54,55] have shown that T2DM RBCs are less deformable and more susceptible to distortion compared to those nondiabetic counterparts. Agrawal et al. [56] showed that T2DM RBCs tend to be larger than normal RBCs. Other researchers [57] discovered that the development of irregularity in the shape of the T2DM RBCs under hyperglycemia would cause a notable loss in cell deformability. In addition, several studies using AFM directly measured the biomechanical properties of diabetic RBCs and confirmed that they are less deformable than normal RBCs (Figure 1).

### 2.1. Simulation Setup

To model the aberrant biomechanics and morphologies of diabetic RBCs, Chang et al. [58] employed a two-component DPD-based RBC model where the cell membrane consists of two distinct components, i.e., the lipid bilayer and the cytoskeleton, and each component is represented by a 2D triangulated network with $N_{v}$ vertices. Additionally, the authors set the normal RBC (N-RBC) surface area, $A_{N-\mathrm{RBC}}$ = 132.87 μm${}^{2}$, cell volume $V_{N-\mathrm{RBC}}$ = 92.45 μm${}^{3}$, and surface area/volume ratio, $A_{N-\mathrm{RBC}}/V_{N-\mathrm{RBC}}$ = 1.44. In general, the two-component DPD-based RBC model is built by capturing the elastic energy, bending energy, bilayer-cytoskeleton interaction energy, and constraints of the constant surface area and enclosed volume. Constraints on the area and volume conservation of RBCs are imposed to mimic the constant area of the lipid bilayer and its incompressible internal fluid. The bilayer-cytoskeleton interaction potential is expressed as the summation of harmonic potentials. More detailed mathematical representations of the cell models can be found in the original paper [58].

It is understood that the membrane elasticity of RBCs describes their resistance to deformation, and membrane viscosity defines the viscous resistance of the cell membrane to shear deformation. Following the analysis by Dao et al. [59], the authors associate the model parameters and the macroscopic elastic properties. The relationship between the bending constant, $k_{b}$, and the macroscopic bending rigidity, $k_{c}$, can be derived as $k_{b}=\left(2/\sqrt{3}\right)k_{c}$ inspired by a similar formula by Helfrich to model a spherical membrane [60].

To determine the actual membrane viscosity, the authors construct a model combining the viscous contributions from both the lipid bilayer and cytoskeleton. In particular, the authors model the normal RBC (N-RBC) with the following parameters: number of vertices in total, $N_{v}$ = 500; RBC shear modulus, $\mu_{0}$ = 4.73 μN/m; RBC bending rigidity, $k_{c}$ = 2.4 ×$10^{-19}$ J; and effective membrane viscosity, $\eta_{m}$ = 0.128 Pa·s. Then, the authors construct three different diabetic RBC models (D-RBC1, D-RBC2, and D-RBC3), based on the existing experimental data, as shown in Figure 1B.

To address the distinguishing experimental data regarding the morphological and mechanical properties of T2DM RBC, the morphology, membrane viscosity, and shear modulus of three aforementioned T2DM RBC models (D-RBC1, D-RBC2, and D-RBC3) are given below, based on existing in-vitro experiments,

(1) $$\begin{array}{c} \mu_{D-\mathrm{RBC}1}/\mu_{N-\mathrm{RBC}}=\mu_{D-\mathrm{RBC}2}/\mu_{N-\mathrm{RBC}}=\mu_{D-\mathrm{RBC}3}/\mu_{N-\mathrm{RBC}}=2\\ A_{D-\mathrm{RBC}1}/V_{D-\mathrm{RBC}1}=A_{N-\mathrm{RBC}}/V_{N-\mathrm{RBC}}=1.44\\ A_{D-\mathrm{RBC}2}/V_{D-\mathrm{RBC}2}=A_{D-\mathrm{RBC}3}/V_{D-\mathrm{RBC}3}=1.04\\ \eta_{D-\mathrm{RBC}1}=\eta_{D-\mathrm{RBC}2}=\eta_{N-\mathrm{RBC}}=0.128\\ \eta_{D-\mathrm{RBC}3}=0.256 \end{array}$$

More detailed mathematical equations for cell mechanical properties can be found in the original paper [58].

### 2.2. Simulation Results

The biomechanics of diabetic RBCs have been studied by performing cell stretching tests, mimicking OT experiments. As shown in Figure 1B, the stretching response of the different normal and diabetic RBC models (N-RBC, D-RBC1, D-RBC2, and D-RBC3) is described by the variety of axial and transverse diameters of the cell. Compared to N-RBC, D-RBC1 shows a significant decrease in axial diameter and a further decrease in axial diameter for D-RBC2. For D-RBC3, the authors observe that the axial diameters are roughly the same as those obtained for D-RBC2. Additional tests on instantaneous fluctuation height of the RBC membrane surface show that the membrane height distributions obtained from D-RBC2 and D-RBC3 remain nearly identical, indicating a trivial effect of membrane fluidity on the cell membrane fluctuations. Furthermore, a stretching-relaxation test of normal and T2DM RBCs using various parameters confirms that the recovery processes are disparate from each other even though all modeled RBCs are able to recover their original shapes.

To approach the effects of cell elasticity and shape on the biorheological behavior of individual T2DM RBCs, the authors simulate the dynamics of T2DM RBCs in a microfluidic channel, where a symmetric converging and diverging nozzle-shaped channel is constructed. As shown in Figure 1C, a lower A/V ratio leads to a decrease in cell transition speed when individual RBCs travel through a 6-μm-wide micro-channel and the increased cell volume (decreased A/V) results in a slow down in the cell passing process. These results show that the increase of flow resistance in diabetic blood is larger than that by the normal blood, hence leading to the significance of the A/V ratio as a determinant of T2DM RBCs traversal across small capillaries. In addition, the authors simulate the tank-treading (TT) motion of a single RBC in Couette flow, i.e., linear planar shear flow. Results in Figure 1D suggest that both D-RBC1 and D-RBC2 have TT motion faster than that of N-RBC. D-RBC2, with an increased cell thickness, maintains even faster rotation, compared to D-RBC1, while D-RBC3 exhibits a slower rotating motion and a longer TT cycle.

## 3. Elevated Aggregation between Diabetic RBCs

**Figure 2:**
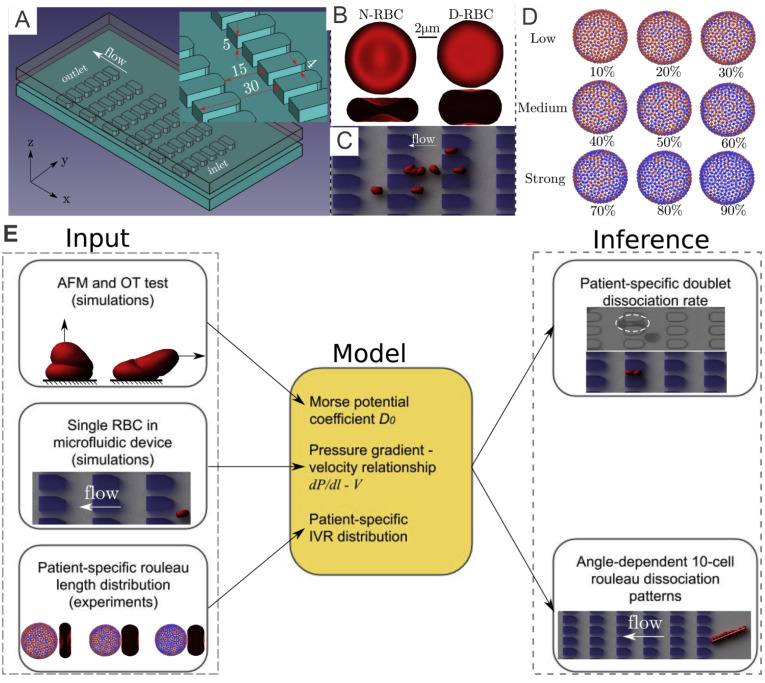
(**A**) A microfluidic device with periodic obstruction is designed for examination of the biomechanics of diabetic RBCs. (**B**) Schematic of a normal RBC (N-RBC) model, which stays as a biconcave shape with surface-to-volume ratio (A/V) of 1.44 whereas a T2DM RBC (D-RBC) model has a more oblate shape with A/V = 1.04. (**C**) An example of simulation results of a T2DM rouleaux travelling through microchannels. (**D**) Fibrinogen-dependent RBC aggregation model where blue vertices denoting “interactive vertices” (IVs) that is able to bind with vertices of the same type located on other RBCs. (**E**) Work pipeline employed to combine in-vivo and in-silico studies. Figures are adopted from [71] with permission.

RBCs suspended in plasma will form a chain-like structure, namely rouleaux, under a low-shear condition. Fibrinogen, as one of the major macromolecules in the plasma, plays a significant role in promoting the cell–cell adhesion between RBCs. Under certain pathophysiological conditions, such as T2DM, exaggerated RBC aggregation leads to enhanced rouleaux formation [67] in the patient’s blood, contributing to vaso-occlusion in small vessels [68,69] and hence preventing the normal oxygen transport in surrounding tissues [70]. In a recent study [71], the authors quantify the fibrinogen-dependent RBC aggregation in diabetic and normal blood. The results suggest that utilizing model parameters guided by microfluidic experiments with the device shown in Figure 2A, researchers can perform patient-specific predictive computational simulations mimicking in-vitro RBC aggregation in the blood flow (Figure 2C) and other in-silico cases (Figure 2E).

### 3.1. Simulation Setup

#### 3.1.1. Normal and Diabetic RBC Models

To investigate the interaction between diabetic RBCs, Deng et al. [71] implement the coarse-grained RBC models based on DPD. The N-RBCs and D-RBCs are represented by a triangulated 2D membrane with 500 DPD particles, see Figure 2B. The morphological and mechanical properties of N-RBCs and D-RBCs follow those in Chang et al. [72].

#### 3.1.2. Mathematical Modeling of Cell-Cell Interaction

The aggregation interaction between RBCs is believed to be a significant component in deciding the size and shape of the RBC aggregates. Fedosov et al. [46] implemented Morse potential to model the intercellular aggregation between RBCs. The Morse potential is given as follows:

(2) $$U_{M}\left(r\right)=D_{0}\left[e^{2\beta (r_{0}-r)}-2e^{\beta (r_{0}-r)}\right],$$

where $D_{0}$ means the depth of the potential well, *e* is the base of the natural logarithm, *r* indicates the distance between two vertices, $r_{0}$ symbolizes zero-force distance and β denotes a distance scaling constant. Specifically, the Morse potential is imposed only on “interactive vertices” denoted by the blue vertices in Figure 2D. Additionally, the repulsive term of the Lennard–Jones (LJ) potential was implemented on all vertices to avoid the RBC membranes from overlapping. The LJ potential is given by

(3) $$U_{LJ}\left(r\right)=4\epsilon \left[{\left(\frac{\sigma }{r}\right)}^{12}-{\left(\frac{\sigma }{r}\right)}^{6}\right],\;\;\left(r\leq r_{\mathrm{LJ}}\right)$$

where $r_{\mathrm{LJ}}$ denotes the cutoff distance, and ϵ and σ denote the scaling factors for energy and distance, respectively.

### 3.2. Results

#### 3.2.1. Diabetic RBC-RBC Detachment at Doublet Level

**Figure 3:**
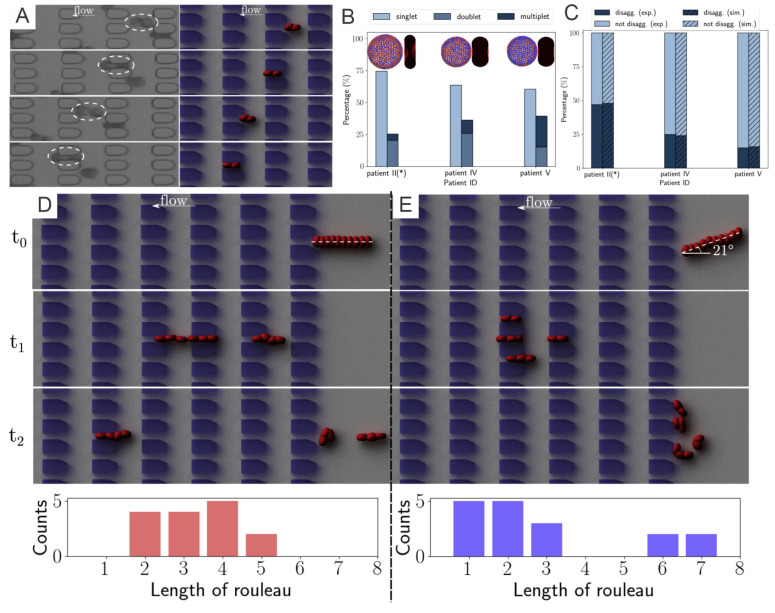
(**A**) Experimental results of a T2DM doublet squeezing through microgates in experiment (left column) and companion simulation (right column). (**B**) Comparison on the percentage of rouleaux with different lengths detected from three different patients (* signifies that patients of obesity, not diabetes). (**C**) Doublet disaggregation rate measured from simulation and experiments; exp., experiment; sim., simulation. Different rouleaux breakup patterns of two 10-cell rouleaux of the rouleaux flow in (**D**) parallel to and (**E**) with a 21${}^{\circ }$ inclination angle to the flow direction ($t_{0}=0\;s,t_{1}\approx 3\;s,t_{2}\approx 4.5\;s$). Bottom histograms show the accumulated results of five different runs. Figures are adopted from [71] with permission.

Based on the results from the corresponding microfluidic experiments that provide in-vitro quantitative knowledge on cell-cell adhesion, see Figure 3A, Deng et al. [71] probe the rouleaux dissociation dynamics at doublet and multiplet (rouleaux consisting of more than two RBCs) levels for patients with or without T2DM. To perform experiment-informed patient-specific simulations, cell–cell interaction models were designed by learning the underlying relationship between the model parameters and rouleaux length distribution. The authors monitor microfluidic experiment videos for each patient and gather two types of statistics for simulations. First, the authors randomly choose 10 snapshots at different time points from the video for each patient, calculate the number of singlets, doublets and multiplets within the region of interest. Then, the authors compute the ratio of each type of rouleaux for patients, see Figure 3B. In addition, from the samples of three selected patients with distinct fibrinogen levels, it is noted that the ratio of singlets, doublets and multiplets vary among patients, by showing a positive correlation to the corresponding fibrinogen concentration levels. The authors then construct patient-specific RBC models from the three groups shown in Figure 2D. To show the overall cell–cell adhesive strength for each patient, the authors plot the averaged “interactive vertices ratio” of patient-specific cell models used in the patient-specific simulations, see Figure 3B.

To validate the experiment-informed RBC model, the authors analyze patient-specific rouleaux dissociation rates at the doublet level; in the experiment, the plain bars in Figure 3C, against those in the simulations, the shaded bars in Figure 3C. The computational and experimental results agree quite well with each other, proving that the distribution of rouleaux length is a strong indicator in doublet breakup rate. Furthermore, these simulations reveal that under the same pressure gradient, T2DM doublets with weak cell–cell adhesion strength easily break up, while doublets with medium cell–cell adhesion strength mostly maintain adhered, and doublets with strong cell–cell adhesion strength are strong enough to remain adhered with high chances. These remarks, consistent with experimental results, further confirm the accuracy of patient-specific RBC models.

#### 3.2.2. Diabetic RBC–RBC Detachment at the Multiplet Level

From the microfluidic experiments performed in [71], the authors notice that the pose of a long rouleaux with respect to the direction of flow significantly affects its later breakup pattern. To understand this phenomenon in greater detail, the authors carry out simulations on two 10-cell rouleaux passing through the microchannels, one along the flow direction (Figure 3D) and the other entering microchannel with a tilted angle to the flow direction (Figure 3E). Specifically, to provide more information regarding the statistics of the phenomenon, the authors run each simulation set for 5 different times and count the rouleaux length, after the rouleaux is broken up by constant collision with the microchannel, see the histogram figures in Figure 3D,E. While rouleaux with a tilted centerline usually break up into many triplets and one quadruplet, rouleaux with centerlines long the flow direction frequently split into only two doublets and two triplets. This finding implies that the angle between long rouleaux centerline and flow direction can tremendously affect the later-on rouleaux breakup patterns. Similar observations were suggested by other studies in the literature [73] that the cross-flow design using various filter shapes and arrangements lead to disparate aggregated cell clogging events, and high efficiency in cell sorting.

## 4. Altered Blood Rheology and Enhanced Platelet Margination in the Diabetic Blood

**Figure 4:**
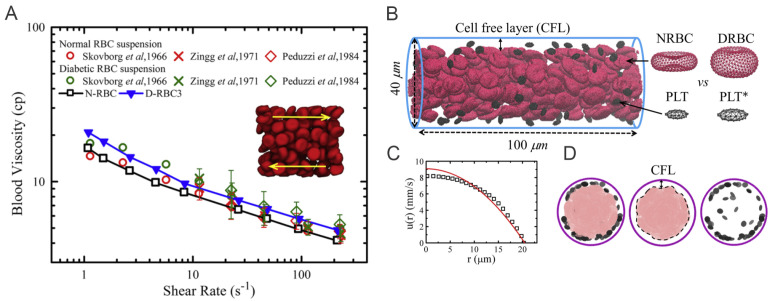
(**A**) Variation of shear viscosity of suspensions of T2DM RBCs with different shear rates at hematocrit Ht = 45%. Circles represent data reported by Skovborg et al. [74]. Crosses represent data reported by Zingg et al. [75]. Diamonds represent data reported Peduzzi et al. [76]. Red symbols represent data computed from normal RBC suspension and green symbols represent data computed from diabetic RBC suspension. Figures are adopted from [58] with permission. (**B**) Side view of experimental setup for modeling the healthy and diabetic blood flow in a cylindrical vessel with diameter D${}_{t}$= 40 μm and hematocrit = 15%. * signifies diabetic platelets. (**C**) A plug-like velocity profile is measured from the blood flow as compared with a parabolic curve (red line). (**D**) Front views of the blood cells in the vessel. Snapshots from left to right illustrate the distributions of RBCs+platelets, RBCs and platelets. Figures (**B**–**D**) are adopted from [72] with permission.

In the last two sections, we have revisited the simulation work on the abnormality of single RBCs or RBC clusters in diabetic blood. Next, we will look into how these abnormalities affect the dynamics of diabetic RBC in blood suspension (Figure 4A) and in blood flows with other blood cells, i.e., platelets and white blood cells (WBCs). Chang et al. [72] combine the properties of diabetic RBCs and platelets from available literature [61,62,63,64] as well as a novel data set collected from 64 diabetic patients. Several hemodynamic factors, such as the blood flow rate, hematocrit, and platelet shape are considered in this work. More importantly, the authors study the influence of WBC dynamics on platelet margination by respectively incorporating WBC flowing, rolling near the endothelial wall, and, finally, firm adhesion to the wall into the computational modeling. Such in-silico studies, comprising a large variety of blood cells, provide precious insights into the transportation and inter-cell dynamics of RBCs, platelets, and leukocytes in diabetic states, which in turn elucidate the complicated relationship between the chronic inflammation and thrombosis in patients with T2DM.

### 4.1. Simulation Setup

#### 4.1.1. Diabetic RBC Suspension under Shear Flow

In the work of Chang et al. [58], the authors implement the same NRBC model as in the previous sections, and use D-RBC3 in [58] to represent the diabetic RBCs. These RBCs are placed between two parallel plates moving in opposite directions to create shear flow under which the viscoisty of the diabetic blood can be measured.

#### 4.1.2. Blood Flow in Cylindrical Channels

To investigate the margination of platelet in the diabetic blood, Chang et al. [72] design the simulation domain as a cylindrical vessel with a diameter of 40 μm. Periodic boundary is implemented along the flow direction, see Figure 4B. A constant body force is applied on each DPD particle to drive the flow and the measurements are conducted after the flow is fully developed, i.e., a plug-like velocity profile as illustrated in Figure 4C. For simulations with adherent WBCs clinically observed in diabetic blood, the WBCs are rolling in the channel, then the contact surface of WBCs are set to be frozen after running to the desired location in the channel.

### 4.2. Results

**Figure 5:**
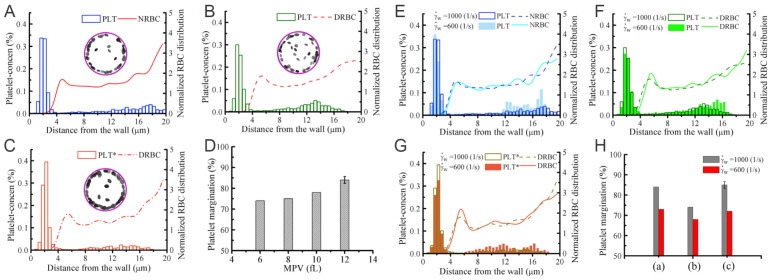
Platelet margination in blood containing healthy and diabetic RBC+platelet. Distributions of platelets and RBCs in the vessel for (**A**) NRBCs+PLTs, (**B**) DRBCs+PLTs (MPV = 6fL), and (**C**) DRBCs+PLT*s (MPV = 12 fL). * signifies the diabetic platelets. (**D**) Effect of MPV of platelets on platelet margination. (**E**–**G**) Effects of flow rate on platelet margination. Distribution of RBCs and platelets in the vessel with cell suspensions of (**E**) NRBCs+PLTs, (**F**) DRBCs+PLTs, and (**G**) DRBCs+PLT*. The percentage of marginated platelets $\phi_{p}$ for all three cases is summarized in (**H**). * signifies the diabetic platelets. Figures are adopted from [72] with permission.

Figure 5A–D compare the difference in local distribution profiles of RBCs and platelets in the channel in case of normal blood and diabetic blood, under the same hematocrit and shear rate. In normal blood flow (Figure 5A), the distribution of RBCs peaked at the centerline following by a secondary peak close to the cell-free layer (CFL). Whereas, in diabetic blood flow (Figure 5B), a relatively lower platelet concentration is discovered in the CFL and a 74% platelet margination rate is observed. It may suggest that the RBC deformability plays an important role in platelet transport, and reduced RBC deformability in diabetic blood may weaken platelet margination. However, when the normal platelet model (mean platelet volume at 6 fL) in Figure 5B is replaced with a platelet model having MPV at 12 fL, the platelet margination pattern changes, see Figure 5C. Therefore, the authors also conduct a comparative study of the impact of the platelet MPV on platelet transport for diabetic blood. The corresponding result in Figure 5D shows that the extent of platelet margination is enhanced with increased MPV in diabetic blood.

The shear rate of blood is another important factor controlling RBC and platelet distributions in a vessel. Chang et al. [72] simulate blood with hematocrit at 20% for three different cell type suspensions: NRBC+PLTs (Figure 5E), DRBC+PLTs (Figure 5F) and DRBC+PLT*s (Figure 5G). The RBC distribution and CFL thickness in Figure 5E show that the normalized RBC distributions do not illustrate notable difference at 1000 s${}^{-1}$ and 600 s${}^{-1}$ whereas a decrease is observed in the platelet concentration within the CFL region in all cases. Comparing Figure 5F,G, the results indicate that the larger platelets are more likely to migrate toward the vessel wall. Figure 5H suggests that there is roughly 6–10% decrease in the platelet margination rate when wall shear rate is decreased from 1000 s${}^{-1}$ and 600 s${}^{-1}$. The weakened platelet margination at lower wall shear rates is probably due to the reduced collision between platelets and RBCs. Moreover, it is also observed that the platelet margination pattern in the case of normal blood flow (Figure 5E) is very similar to diabetic blood flow with larger MPV (Figure 5G), and both have higher platelet margination rates compared with diabetic blood with normal MPV (Figure 5F).

## 5. Exacerbated Platelet Aggregation in Diabetic Blood Flow

Continuum-based partial differential equation (PDE) models [77,78,79,80,81,82,83,84] have been broadly used to describe the time and spatial dependence of thrombin generation, fibrin formation, and thrombus growth under various flow conditions. However, simulations of the development of thrombosis in diabetes require discrete representation of the blood flow at cellular level in the computational models. In order to describe the microscale interactions between blood cells, numerous computational models [85,86,87,88,89,90,91,92,93,94,95] have been developed to along this direction, see reviews in [77,96]. Particularly, hybrid models, which couple PDE models with particle-based models using heterogeneous solvers [93,95,96,97,98], such as integration of finite element method with DPD [88] or integration of FEM, lattice boltzmann and lattice kinetic Monte Carlo [99], have been developed to simulate platelet activation and aggregation by treating platelets as particles. However, applications of these hybrid models are mostly limited to two-dimensions due to the model complexity and the ensuing significant computational cost.

### 5.1. Simulation Setup

#### 5.1.1. Simulation Methodology

**Figure 6:**
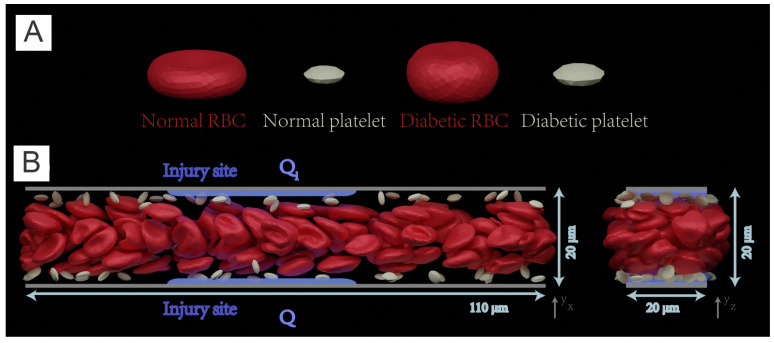
(**A**) Normal and diabetic RBC and platelet models, validated in Chang et al. [58], are employed to simulate blood flow in (**B**) a 3D rectangular microchannel of size of 110 μm × 20 μm × 20 μm. Left: front view, right: side view. Solvent particles in the simulate carry 22 species for modeling the coagulation cascade that is initiated from two injury sites (highlighted with blue color lines) with a size of 40 μm located at the upper and bottom channel wall respectively. Figure is reproduced from [104].

In this part, we will review a recent multiscale numerical framework by Yazdani et al. [100] that combines the four key sub-processes of blood clotting—namely hydrodynamics, cell mechanics, transport of coagulation factors and coagulation reactions—such that it can be used to investigate the contributions of multiple prothrombotic factors induced by T2DM. In this work, normal and diabetic RBC and platelet models (Figure 6A) are employed to simulate normal and diabetic blood flowing in a rectangular channel. The platelet, represented by a nearly rigid ellipsoid, is extended from [72,100]. This multiphysics and multiscale framework was built on a single computational platform (LAMMPS), thereby significantly reducing the computational cost induced by the communication overhead between different solvers. In this work, the authors employ the normal or diabetic RBCs model validated in the previous work of [22,23,71,101,102,103].

Transport of coagulation factors in the coagulation cascade is simulated by transport DPD (tDPD) [105] that can account for concentration fields. To consider the coagulation cascade, the authors implement the mathematical model of Anand et al. [106], which describes the coagulation pathways using a set of 21 coupled advection–diffusion reaction equations involving 23 factors.

The author employed a stochastic bond formation–rupture model proposed by Mody and King [107] to consider the adhesive dynamics of receptors on the platelet membrane binding to their ligands. A detailed description of the adhesive dynamics model can be found in the original paper [104].

#### 5.1.2. Simulation Domain

The simulations are performed in a 3D rectangular microchannel with a size of $110\times 20\times 20\;\mu m^{3}$. The channel is prefilled with RBCs, platelets and solvent particles as illustrated in Figure 6B. Periodic boundary conditions are employed in the *x* and *z* directions. The adhesion and aggregation of platelets are examined in normal and diabetic blood, respectively. Initially, platelets are intentionally placed close to the upper and lower sides of the channel such that th emargination of platelets can be quickly achieved.

### 5.2. Results

#### 5.2.1. Platelet Adhesion and Aggregation in Normal Blood and Diabetic Blood

Before the main simulations capture the coagulation cascade, a warm-up simulation is performed to allow the blood flow become fully developed. A constant body force is applied to each DPD particle to drive the blood flow, yielding a plug-like velocity profile in the microchannel. The characteristic shear rate, defined as the wall shear rate of a parabolic Poiseuille flow with the same mean velocity, is selected to be $\dot{\gamma_{\omega }}=6\bar{u}/H$ = $615\;s^{-1}$.

**Figure 7:**
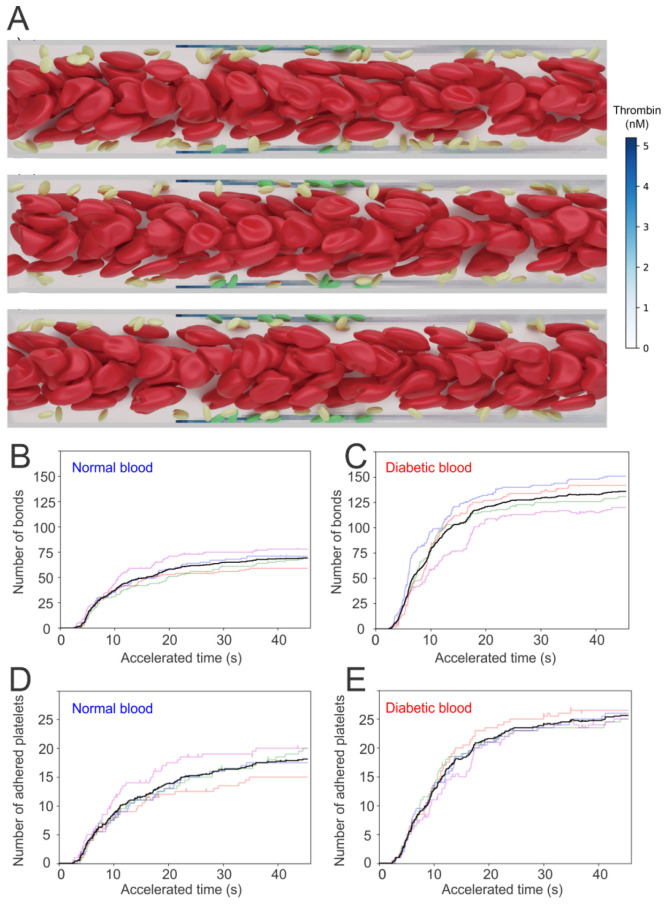
(**A**) Simulation results of normal RBCs and platelets moving in the microchannel. Activated platelets adhere to the injury sites after they become activated by thrombin with concentration >1 nM. The number of bonds generated between platelets and injury sites for (**B**) normal and (**C**) diabetic blood as well as the number of adhered platelets for (**D**) normal and (**E**) diabetic blood are recorded for 40 s. Figure is reproduced from [104].

Next, the coagulation cascade is initiated by injecting the four factors into the flowing blood from the injured sites. A threshold value of 1 nM is selected for thrombin-controlled platelet activation, following the work of [108]. The platelets moving into the boundary layer with [IIa] > 1 nM are instantaneously turned into activated mode, which allows platelets to form bonds with the particles placed at the site of injury. Figure 7A presents three snapshots illustrating the sequential processes of platelets’ activation and adhesion to the site of injury (highlighted with green particles).

Quantification of the adhesion dynamics between the platelets and the injured vessel is considered by recording the total number of bonds formed between activated platelets and the ligands that represent the Von Willebrand factor(vWF) at the injury sites—see Figure 7B,C for normal and diabetic blood, respectively. To quantify the uncertainty of the simulations, five independent simulations are performed under the same hemodynamic condition, but with different stochastic realizations. The numbers of platelets that are adhered to the injury sites with respect to time are also recorded. Figure 7D,E illustrates that the number of bonds formed between the platelets and injury sites, as well as the number of the adhered platelets, begin to increase drastically after an initial time lag of approximately 5 s. In particular, the increase of these numbers computed from diabetic blood is greater than those of normal blood.

#### 5.2.2. Fibrin Kinetics Comparison between Normal Blood and Diabetic Blood

**Figure 8:**
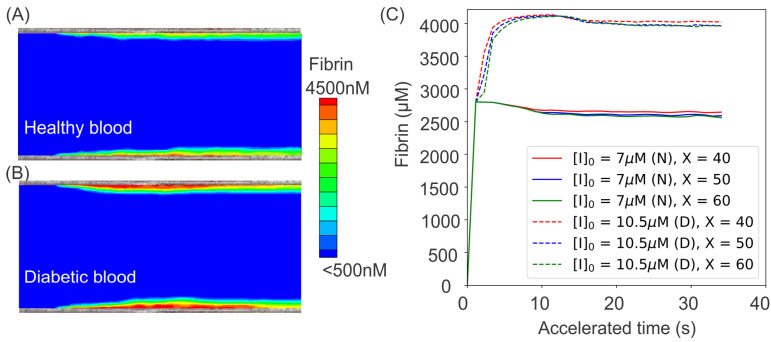
Simulation results of fibrin ([Ia]) concentration boundary layers in the middle cross-section of the microchannel for (**A**) normal and (**B**) diabetic blood. (**C**) Variation of fibrin concentration over time at three different sites of x=40,50,60μm for normal blood (solid lines) and diabetic blood (dashed lines). To investigate the effects of diabetic blood in promoting fibrin generation, an initial concentration of [I]${}_{0}$ = 7 μm is prescribed in normal blood model while [I]${}_{0}$ = 10.5 μm is used in the diabetic blood model. (N) represents normal blood whereas (D) designates diabetic blood. The figure is reproduced from [104].

In addition to examining the effect of the altered biomechanics of blood cells in promoting platelet aggregation, the authors of [104] also investigate the impact of abnormal coagulation factors in the diabetes on exacerbating thrombus formation. Following the study of [109], the authors select the concentration of plasma fibrinogen, a key source of fibrin in the circulating blood, to be 50% higher than normal blood. The authors also assumed that a fibrin concentration of 350 nM represents the boundary of a growing thrombus. The simulation results in the cases of normal and diabetic blood are shown in Figure 8A,B, which demonstrate that the excessive fibrinogen in diabetic blood could result in a greater level of clot formation at the injury areas on the vessel wall. The generation of fibrin at the injury sites ia further quantified in Figure 8C, which shows that the extent of fibrin generation at the injury sites in diabetic blood is as much as 45% greater than that of normal blood because of the excessive fibrinogen.

## 6. Perspective

Computational modeling has emerged as a powerful tool for investigating pathological processes in many blood diseases. Although the computational modeling of RBCs in diabetes has improved our understanding of the adverse effects of the abnormal biomechanics and biorheology of diabetic blood and their connections to clinical manifestations, there are still many open areas that could be targeted as future research areas, including but not limited to:

(1) Modeling diabetic RBCs at the molecular level. The underlying molecular mechanisms that cause the abnormal shape and aberrant biomechanical properties of diabetic RBCs is still elusive. Elucidation of the biological processes that lead to pathological alterations of diabetic RBCs requires detailed simulation at the protein and sub-cellular levels. In the last two decades, RBC models with protein-level representations have been developed to investigate the underlying processes associated with RBCs’ lipid membrane, cytoskeleton defects, and remodeling—see recent reviews [101,102,103,110]. Protein-level RBC models, such as in [25,111,112,113], have also been used to assess the altered mechanical properties and morphologies of RBCs induced by either protein defects in blood disorders or virus invasion [114,115,116,117,118,119,120]. Thus, molecular-level simulations can be potentially used to investigate the causes of the changes of RBCs’ morphologies and biomechanics, thereby facilitating the discovery of new therapeutic treatment to improve blood circulation.
(2) Organ-specific modeling of diabetic blood in microvasculature. In addition to cardiovascular disease, the altered biomechanics of diabetic RBCs could contribute to other complications in T2DM, such as neuropathy (nerve damage), nephropathy (kidney damage), retinopathy (eye damage), foot damage and so on. For example, diabetic retinopathy (DR), is the most common microvascular complication of diabetes. Microaneurysm (MAs), a type of lesions occurring in the microvasculature of the retina, are one of the earliest clinically visible signs for diagnosing DR [121]. The rupture of MAs may lead to in retinal edema or hemorrhage, which can directly affect retinal function. Although microfluidic experiments have been performed to investigate blood cell transport and hemodynamics in the MAs [122], unfolding some biological processes and testing the associated hypotheses that involve blood cell interactions still requires complementary computational simulations [123]. For example, the enhanced cell adhesion in diabetic blood could promote RBC aggregation and enlarge the cell-free layer near the vessel walls [124,125,126], causing reduced average hematocrit level in the branching vessels. As a result, the average hematocrit of the blood in capillaries is lower in diabetic patients than in normal subjects, causing hypoxia within te microvasculature [70,127,128]. Microvascular hypoxia of the retina could trigger the leaking or rupture of MAs, thereby elevating the severity of the disease. Thus, computational studies of diabetic blood flow in organ-specific structures could provide insights into the mechanisms of organ damage from the hematological perspective.
(3) Bridging glucose levels with blood rheology in diabetes using artificial intelligence (AI). Advanced machine learning techniques have been widely used to predict short-term glucose levels within one hour, including purely data-driven models making use of historical glucose levels [129,130,131], as well as other physiological models utilizing both past glucose levels and auxiliary information [132,133], such as meal intake, exercise intensity, and insulin injection. However, there is lack of models to connect the prediction of glucose level with blood rheological changes, which can be used to assess the risks for developing thrombotic events based on patient-specific data. We believe that it is of great importance to leverage the advantages in both bioinformatic modeling and biomechanistic modeling, by using bioinformatic modeling tools to provide patient-specific biomechanistic modeling parameters. In return, patient-specific biomechanistic modeling could predict disease progression to supply more synthetic data for bioinformatic modeling. We hope that the modeling cycle will benefit the patients with prognostic analyses and beneficial treatment advice.

## Data Availability

Not Applicable.
